# Increased Risk of Parental Instability for Children Born Very Preterm and Impacts on Neurodevelopmental Outcomes at Age 12

**DOI:** 10.3390/children9030304

**Published:** 2022-02-23

**Authors:** Megan E. Gath, Samantha J. Lee, Nicola C. Austin, Lianne J. Woodward

**Affiliations:** 1 Child Well-Being Research Institute, Faculty of Education, University of Canterbury, Christchurch 8011, New Zealand; megan.gath@canterbury.ac.nz (M.E.G.); samantha.lee@canterbury.ac.nz (S.J.L.); 2 Faculty of Health, University of Canterbury, Christchurch 8041, New Zealand; 3 Canterbury Child Development Research Group, University of Canterbury, Christchurch 8041, New Zealand; 4 Christchurch Women’s Hospital, 2 Riccarton Ave, Christchurch 8011, New Zealand; nicola.austin@cdhb.health.nz; 5 Department of Paediatrics, University of Otago, Christchurch 8011, New Zealand

**Keywords:** preterm, very low birthweight, parental change, neurodevelopment, inter-partner relations

## Abstract

Caring for a child born preterm places significant emotional and financial burdens on family relationships. This paper examines (a) the extent to which children born very and extremely preterm are more likely to experience parental change/caregiver instability than children born full term, (b) predictors of parental change/s for preterm infants, and (c) whether exposure to parental change/caregiver instability increases child neurodevelopmental risk. Data were collected as part of a prospective longitudinal study of 110 very preterm and 113 full-term born infants and their parents studied from birth to corrected age 12 years. At ages 2, 4, 6, 9 and 12 years, detailed information was collected about the frequency and nature of all parent/caregiver changes for 3–6 monthly intervals of each child’s life. At age 12, all children completed a comprehensive neurodevelopmental evaluation of their emotional and behavioural adjustment, cognition, and educational achievement. Results showed that children born very preterm were at increased risk of experiencing parental/caregiver changes, with this risk being greatest for those born extremely preterm. Neonatal medical complexity, family socioeconomic disadvantage, maternal psychological wellbeing, and child neurodevelopmental impairment were associated with a higher risk of parental change. Preterm birth and exposure to parental change/instability contributed additively to poorer child outcomes. Findings support the need for family-focused neonatal and postnatal care strategies for high-risk infants, to support parents as well as their infants to optimize child health and developmental outcomes.

## 1. Introduction

Becoming the parent of a premature infant is a highly stressful life event for families [1,2]. Not only do these parents have to cope with the loss of a normal birth and parenting experience, but their infants are often critically unwell and admitted to the neonatal intensive care unit (NICU) resulting in an extended period of parent–infant separation. Mounting evidence shows that these experiences have a significant impact on parental mental health and family life during their infant’s NICU stay [3,4,5,6]. For example, mothers of very preterm (VPT; <32 weeks gestation) infants report higher levels of psychological distress than mothers of full term born infants, with between 25–50% reporting clinically significant levels of anxiety and depression [3,4,5]. Although data on fathers is limited, preliminary findings also suggest fathers also experience heightened distress after a VPT birth [6] and early involvement of fathers is an important influence on neurodevelopment in medically at-risk neonates [7].

Furthermore, these challenges and stressors do not end at the hospital door. Children born VPT are at high risk for a range of health and neurodevelopmental impairments including feeding, respiratory and other health issues, more frequent hospitalisations, cerebral palsy, cognitive delay, executive dysfunction, and emotional and behavioural adjustment problems [8,9,10]. Comorbidity is also common, with at least a third subject to multiple neurodevelopmental domain impairments that likely worsen everyday child and family difficulties, as well as longer-term neurodevelopmental prognosis [11,12]. Yet relatively little is known about the longer-term impacts of raising a VPT born child on family functioning, and in particular the quality of inter-partner relations and the extent of marital/partner relationship stability. This is surprising given data showing that parents raising children with health and disability issues report poorer marital quality and are at increased risk of separation/divorce than parents of typically developing children [13,14,15,16]. Additionally, raising a VPT born infant has substantial economic impacts on families, due to ongoing health and education service needs [17], as well as possible adaptations to parental work schedules and living conditions to accommodate their child’s additional developmental needs [18,19]. All of these factors may negatively affect the relationship between parents and increase the risk of family breakdown.

### 1.1. Impact of Preterm Birth on Inter-Partner Relationship Stability

There is some research to support the adverse impacts of preterm birth on family functioning, including disrupted social relationships, parental coping difficulties and increased financial costs particularly during early childhood (see Treyvaud [20] for a review). In general, findings suggest that the impacts are greater when infants are born at lower gestational ages/birth weight or experience higher neonatal medical risk. There is also some suggestion that family effects may be greatest during early childhood and decrease over time as children reach adolescence [21,22].

To date, only two studies have examined the effect of VPT birth on the risk of parental separation/divorce. First, a short-term retrospective study using data from the 1988 National Maternal and Infant Survey (*n* = 6016) found that by age 2, parents of very low birth weight (VLBW; <1500 g) infants were two times more likely than parents of higher birth weight (>1500 g) infants to have separated or divorced [23]. Extending on this work, a prospective population-based cohort study of 5732 preterm births (<35 weeks gestation) in France found that by age 7, 10% of parents with a preterm infant had separated. Child neurodevelopmental impairment at age 2 fully mediated associations between preterm birth and subsequent separation risk [24]. A further follow-up of 3300 of these children found that, independent of family socioeconomic status, preterm infants whose parents had separated by age 5 were more likely to be rated by their teachers on a global measure of school functioning as having more academic and behavioural difficulties in the classroom than those whose parents had not separated [25]. This analysis did not include a control group, so could not assess the extent to which rates of parental separation and school problems differed relative to typically developing term-born children.

Whilst informative and helpful in highlighting the need to consider, both in the NICU and after discharge, the effects of preterm birth on partner/marital relations, it is important to note that existing studies were relatively short term, provided limited to no information about the possible additive/interactive effects of family breakdown/parental change on the neurodevelopmental outcomes of children born very preterm. These issues are important given clear evidence from general population studies showing that parental separation and change can exert lasting impacts on children across the lifespan, including increased risk of school drop-out, behaviour problems, poor academic achievement, worsened physical health, and increased risk of partnership instability in their own adult intimate relationships [26,27,28,29].

To address these important research gaps, we examined relations between very preterm birth and parental change/instability in a longitudinal study of children and families from birth to age 12 years. We also examined the neonatal medical, family psychosocial, and child factors that placed VPT born children at increased risk of experiencing parental change/instability, and assessed the impact of parental change/s on child neurodevelopmental outcomes over and above, or in combination with, the risks associated with preterm birth. For the purposes of this paper, we use the term “parental change/instability” to refer to transitions in the family unit that alter a child’s parent or caregiver figures within their primary home. This includes, but is not limited to, parental separation and divorce, formation of new partner relationships and the dissolution of those relationships, parental death, and/or child placement into adoption or foster care.

### 1.2. Aims

To document the extent of parental change experienced by children born very preterm relative to children born full-term from birth to age 12 years, with a particular focus on whether exposure to parental change varied with infant gestational age at birth.

To identify neonatal medical, family psychosocial, and child factors that place very preterm born children at an increased risk of exposure to parental change/instability from birth to age 12 years.

To examine the extent to which childhood exposure to parental change/instability has an additive and/or interactive effect on very preterm born children’s risk for a range of adverse neurodevelopmental outcomes relative to term-born children.

## 2. Materials and Methods

### 2.1. Design and Participants

Data were drawn from a prospective longitudinal investigation of the effects of preterm birth on children’s neurodevelopmental outcomes. Study participants consisted of two groups of children and their parents. Exclusion criteria across all groups included infant congenital abnormality and non-English speaking mothers.

#### 2.1.1. Very Preterm Group

The first group consisted of an unselected regional cohort of 110 children born VPT (≤32 weeks gestation) and their mothers. These children were admitted consecutively to the level III Neonatal Intensive Care Unit at Christchurch Women’s Hospital, New Zealand, between November 1998–December 2000. Ninety-two percent of all eligible infants were recruited. Excluding infant deaths (*n* = 3), 96% (*n* = 103) of families were successfully followed up to 12-years corrected child age.

#### 2.1.2. Full Term Group

The second group comprised a comparison sample of 113 FT-born (37–41 weeks gestation) children and their mothers recruited at age 2 years. Full-term comparison children were identified from the delivery schedule of Christchurch Women’s Hospital by identifying, in an alternating manner, a child of the same sex who was born two deliveries prior to, or following, the birth of each very preterm born child. Sixty-two percent of those identified and contacted were successfully recruited. Reasons for nonparticipation included untraced (*n* = 32), relocated overseas (*n* = 9), declined participation (*n* = 9), and agreed but could not attend clinic appointment in required age/time window (*n* = 19). No significant differences were found between recruited and non-recruited term-born infants and their families in terms of gestational age, birth weight, socioeconomic status (SES), or ethnicity (*p* > 0.05). In addition, comparison of the socioeconomic profile of recruited families of full-term born children with regional census data showed that they were highly representative of the Canterbury region from which they were drawn [30]. Sample retention of the full-term group was also high, with 96% (*n* = 109) of families followed up to child age 12 years.

#### 2.1.3. Current Study

There were 19 sets of twins in our sample of children (2 sets of full-term twins and 17 sets of preterm born twins). As the current analyses were focused on the family unit, one twin from each twin pairing was randomly excluded. After excluding 19 children (one twin from each set), we had a total of 197 children remaining in our total sample (*n* = 111 full term, *n* = 86 VPT). For the purposes of this analysis, the VPT group was further stratified by infant gestational age into VPT (≤32 weeks and ≥28 weeks, *n* = 48) and EPT (<28 weeks, *n* = 38) to examine the effect of the extent of prematurity on parental separation/change risk. Table 1 describes the characteristics of these three study groups.

### 2.2. Procedure

All study families were contacted to take part in five follow-up study waves within 2–4 weeks of their study child’s 2nd, 4th, 6th, 9th, and 12th birthdays (corrected for gestational age at birth). Mothers or primary caregivers completed a semi-structured interview administered by a trained interviewer at each follow-up assessment. Then, at 12-years corrected age, all children completed a comprehensive neurodevelopmental assessment administered by a clinical psychologist and supervised postgraduate level research assistants who were blinded to birth group and perinatal history. A parent interview was also completed by an unblinded research coordinator. All procedures and measures were approved by the Upper South Regional Ethics Committee, and at each follow-up wave written informed consent was obtained from all parents or guardians.

### 2.3. Measures

#### 2.3.1. Parental Instability

At each follow-up assessment, using life history calendar methods, mothers were asked to report on their study child’s living arrangements and family composition as well as any caregiver changes that occurred for 3/6 monthly intervals since the last assessment. Qualitative details regarding each change were recorded and later coded into the following nine categories to describe the nature of each reported parental change: parental separation; parental reconciliation; child adopted or fostered; parental death; a new parental relationship; the breakup of a parental relationship; parental imprisonment; child placed in social welfare custody; or other, such as loss of contact with a non-custodial parent or change of custodial parent. A variable indicating the total cumulative number of parental changes children had experienced by each follow-up assessment age was then computed.

#### 2.3.2. Infant Medical Factors

Gestational age at birth was collected through hospital records. In addition, a medical complexity indicator was created by identifying five major medical exposures that complicated an infant’s medical course and potentially posed additional stress for parents and families. These included respiratory support, neonatal sepsis, major surgery, time on parenteral nutrition, and intraventricular haemorrhage/periventricular leukomalacia (IVH/PVL). As identified by Hunt [31], the need for major surgery was an important addition to our complexity parameters, which along with the more frequent variables IVH/PVL, sepsis and moderately severe chronic lung disease are recognised to have a significant impact on neurodevelopmental outcomes [32]. Infants were classified into two groups, those subject to any of these medical challenges and those not exposed to any of these neonatal factors. Of children born preterm, 38.7% had no neonatal medical complexity and 61.3% were subject to at least one form of medical complexity.

#### 2.3.3. Family Psychosocial Factors

Three measures of family psychosocial characteristics were included. The first was maternal age at childbirth.

Second, family socioeconomic status was measured when children were 2 years of age using the Elley-Irving Socioeconomic Index. Parental occupations were coded into one of seven categories: 1–2 = professional/managerial roles, 3–4 = technical/skilled work, 5–6 = semi- and unskilled work, and 7 = unemployed. One code was assigned to each family, based on the occupation representing the lower SES code and therefore the higher SES rating.

Third, multiple measures of maternal mental health and well-being were included. Maternal anxiety and depression was assessed at every age using the Hospital Anxiety and Depression Scale (HADS) [33]. The HADS is a 14-item scale, with seven items each assessing anxiety and depression symptomology. Items are self-rated on a 4-point scale from *Not at all* (0) to *Most of the time* (3). The HADS is a brief and reliable measure (e.g., mean Cronbach’s alpha > 0.80 across 15 studies) with good sensitivity (0.78, 0.79) and specificity (0.90, 0.83) for diagnosing anxiety and depression, respectively [34].

Maternal parenting stress was assessed at ages 2, 4, 6, and 9 using the Parent Distress subscale of the Parenting Stress Index, short form (PSI-SF) [35]. This subscale contains 12 items assessing the stress and distress mothers face as a result of having children. Example items include: “*I feel trapped by my responsibilities as a parent*” and “*I often feel that I cannot handle things very well*”. Items are rated on a 5-point scale from *Strongly disagree* to *Strongly agree.* The 12 items at each age were then summed to create an overall measure of parenting stress. Cronbach’s alpha ranged from 0.83 to 0.88 across assessment points. As parenting stress was not assessed at age 12, PSI scores at age 9 were used as the predictor at age 12.

An overall score of concurrent maternal mental health was calculated at each assessment age as the sum of standardised HADS depression, HADS anxiety, and PSI parenting stress scores.

#### 2.3.4. Child Neurodevelopmental Impairment during Early and Middle Childhood

A composite measure of child neurodevelopmental functioning/impairment was created at each age to indicate the number of co-morbid impairments. At age 2, this composite included one point for each of the following impairments: cerebral palsy diagnosis, any behavioural adjustment difficulties in the clinical range (SDQ), significant delay on the Bayley II Mental Development Index (>2 SDs) [36], and significant delay on the Bayley II Psychomotor Development Index (>2 SDs). At age 4, this composite included one point for each of the following impairments: cerebral palsy diagnosis, language impairment (assessed using the Clinical Evaluation of Language Fundamentals) [37], cognitive delay (assessed with the Wechsler Preschool and Primary Scale of Intelligence WPPSI-R) [38], and any behavioural adjustment difficulties in the clinical range (assessed with the Strengths and Difficulties Questionnaire (SDQ) [39]. At ages 6, 9, and 12, impairments included were: cerebral palsy or developmental coordination disorder, motor function impairment (assessed with the Movement Assessment Battery for Children M-ABC) [40], cognitive delay (assessed with the Wechsler Intelligence Scale for Children WISC) [41], any educational delay (assessed with the Woodcock Johnson-III Tests of Achievement) [42] and any behavioural adjustment difficulties in the clinical range (SDQ).

#### 2.3.5. Child Outcomes at Age 12 Years

Emotional and Behavioural Adjustment was measured using the teacher-reported Strengths and Difficulties Questionnaire (SDQ) [39]. The SDQ is a 25-item screening measure of emotional symptoms, hyperactivity/inattention, conduct problems, peer problems, and prosocial behaviour. Items in each of these subscales are scored on a 3-point scale (0 = not true, 1 = somewhat true, 2 = certainly true) and, with the exception of Prosocial items, are summed to provide a Total Difficulties Score. The SDQ is a widely used measure with good internal consistency (α = 0.82) and test–retest reliability (*r* = 0.84) for the teacher-rated total difficulties scores [43].

Attention-Deficit/Hyperactivity Disorder (ADHD) Symptomology was measured using the 18-item Strengths and Weaknesses of Attention-Deficit/Hyperactivity Disorder Symptoms and Normal Behavior Scale (SWAN) [44]. Mothers reported how their child compared to other children of the same age, based on their observations over the past month. Items were rated on a 7-point scale from *Far below average* to *Far above average*. Higher scores reflected greater symptomology. Sample items include: “*Sustain attention on tasks or play activities*”, “*Follow through on instructions and finish school work/chores*”, and “*Engage in tasks that require sustained mental effort*”. The SWAN has high internal consistency (over 0.90 for both the Inattention and Hyperactivity/Impulsivity subscales), and has good test–retest reliability [45,46].

General Cognitive Ability was assessed using a short form of the WISC-IV [41]. Children completed the block design, similarities, coding, vocabulary, and arithmetic subtests, with composite scores used to calculate an estimate of FSIQ. Two VPT children who were unable to complete the WISC-IV due to severe disability were assigned an IQ score of 40. Abbreviated assessments of the WISC-IV have been demonstrated to be valid measures of general cognitive ability (coefficients exceed 0.90) and are commonly used in research and practice [47].

Educational Achievement was measured using the Australian adaptation of the Woodcock Johnson-III Tests of Achievement (WJ-III) [42]. Children’s standardized Broad Reading and Broad Math scores were calculated from their performance across the following six subtests: Letter-Word Identification, Reading Fluency, Passage Comprehension (reading skills), and Calculation, Math Fluency, and Applied Problems (mathematical skills). These subtests have high internal consistency, with Cronbach’s alpha ranging from 0.83–0.92 [41]. The WJ-III also has good concurrent validity, correlating well with other validated tests of achievement [48].

### 2.4. Statistical Analyses

Analyses were performed using SPSS Version 26. Chi-square tests for independence were used to examine between-group differences in the number of parental changes overall. Logistic regression analyses were then used to examine, within families with children born preterm only, neonatal medical and psychosocial predictors of parental instability. Finally, analyses of variance were used to predict socioemotional, behavioural, and educational outcomes at age 12 from children’s prematurity status (preterm vs. full term) and history of parental instability. Interaction effects between gestational age and parental instability were also examined.

## 3. Results

### 3.1. Nature and Extent of Children’s Exposure to Parental Change from Birth to Corrected Age 12 Years in the Total Sample

**Figure 1:**
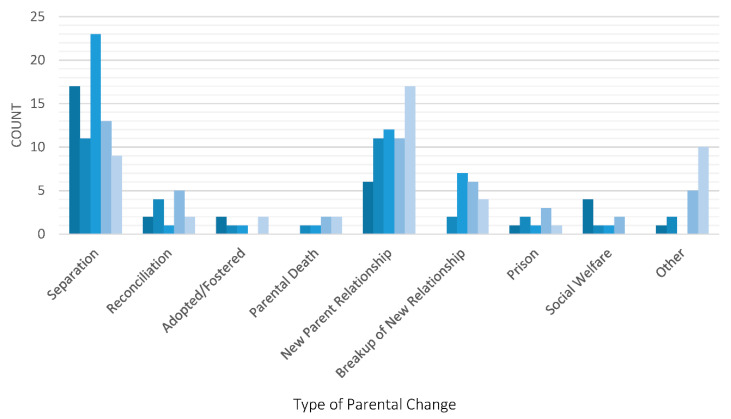
Types of parental instability by age.

Examination of the total cumulative number of parental changes experienced by age 12 showed that more than half of all children (62.5%) had experienced no parental changes by age 12. The highest number of cumulative parental changes experienced was 9. Figure 1 presents the different types of parental changes experienced by all study children in the cohort and the age at which these changes occurred. The most common reason for a parental change was parental separation, followed by the formation of a new partner relationship. Parental separation was more common at younger than older ages and was particularly high during the first two years following birth and between ages 4 to 6 years. The formation of new parental relationships increased in frequency over time, as well as the frequency of “other” changes which primarily consisted of alterations to children’s custodial circumstances, such as care being transferred back to a previously non-custodial parent.

### 3.2. Impact of Preterm Birth on Parental Instability

The first aim of our research was to examine the effects of infant gestational age at birth on subsequent parental change/instability risk. Due to the skewed nature of the parental change data, for the remaining analyses, parental change data was re-scaled in the following ways: the presence of *any parental change/instability* (1 or more parental changes) and the presence of *high levels of parental change/instability* (2 or more parental changes).

Table 2 shows the percentage of children born full term (FT, 37+ weeks), very preterm (VPT, ≤32 and ≥28 weeks), and extremely preterm (EPT, <28 weeks) who experienced (a) any and (b) two or more (multiple) parental changes from birth to corrected age 12 years.

Table 2 presents the rates of parental change by age, and we next used chi-square analyses to tested overall differences (across ages) in risk of both any and multiple parental changes. With respect to any parental change, EPT children were significantly more likely to experience at least one parental change than FT (χ^2^(1) = 16.34, *p* < 0.001) and VPT born children (χ^2^(1) = 12.86, *p* < 0.001). Specifically, as shown in Table 2, by age 12 almost half of EPT-born children had experienced at least one parental change compared to just over a third of FT and VPT born children. Overall, EPT children were 1.7 times more likely to experience any parental change than children born full term. No significant differences were found between FT and VPT children in their risk of exposure to a parental change (χ^2^(1) = 0.04, *p* = 0.85).

**Figure 2:**
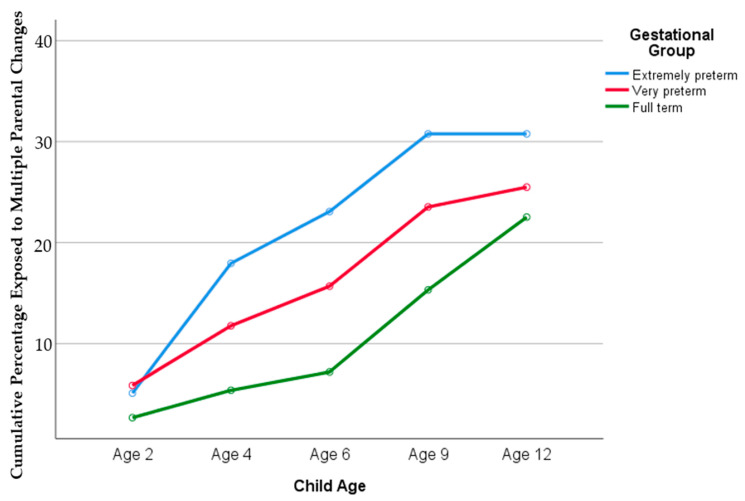
Percentage of children who have experienced two or more parental changes by gestation group.

In contrast, there was a general trend for the risk of exposure to multiple parental changes over time/age to increase with decreasing gestational age (see Figure 2). Both EPT and VPT born children were significantly more likely than FT children to experience multiple (2 or more) parental changes, with EPT children being two times more likely (χ^2^(1) = 14.73, *p* < 0.001) and VPT born children being 1.6 times more likely than FT children to experienced multiple parental changes by age 12 (χ^2^(1) = 5.46, *p* = 0.02). The difference between EPT and VPT children’s risk of multiple parental changes did not reach statistical significance (χ^2^(1) = 1.87, *p* = 0.17).

To illustrate these findings, Figure 2 shows the cumulative percentage of children experiencing 2 or more parental changes over time by the gestational group. The figure clearly illustrates the increasing risk of multiple parental changes with age, and that risk is heightened for those born at lower gestational ages.

### 3.3. Predictors of Parental Change in Families of Children Born Preterm

Our second aim was to identify predictors of any parental change in families with a child born preterm. For this analysis, we focused on the subsample of VPT and EPT children (*n* = 86) only. Logistic regressions were used to predict whether or not children had experienced any parental change at each age from the following medical, family psychosocial, and child risk factors: gestational age in weeks at birth, neonatal medical complexity, family socioeconomic status, maternal age at birth, concurrent maternal mental health and concurrent child neurodevelopmental impairments. Thus, our predictors included both time-invariant (e.g., maternal age at birth, neonatal medical complexity) and time-varying (e.g., maternal mental health, child functioning/extent of impairment) risk factors.

The overall binary logistic models were significant at every age [likelihood ratio tests: Age 2: χ^2^ (6) = 16.17, *p* = 0.01; Age 4: χ^2^ (6) = 16.69, *p* = 0.01; Age 6: χ^2^ (6) = 37.44, *p* < 0.001; Age 9: χ^2^ (6) = 16.97, *p* = 0.001; Age 12: χ^2^ (6) = 29.60, *p* < 0.001]. Odds ratios and significance levels for the predictors in each model are presented in Table 3.

Earlier gestational age and child neonatal medical complexity were significant predictors of instability at later ages; however, the large odds ratio for medical complexity at age 2 was close to significant (*p* = 0.08) and was likely not found to be significant due to the small number of children who had experienced a parental change by age 2.

Low socioeconomic status was a predictor of increased risk of instability within families of preterm born children across all ages. Maternal mental health was also a predictive factor at ages 6 and 12, with increased symptoms of depression, anxiety, and parental distress associated with greater instability. Concurrent assessments of children’s neurodevelopmental impairments were a significant predictor of parental instability only at age 2. When examining predictors of multiple parental changes, the effects were generally similar although had low power due to the smaller number of children experiencing multiple parental changes. These findings generally suggest that in addition to child gestational age at birth, infant medical complexity and family social circumstances contribute to subsequent family breakdown risk, but potentially with age-specific effects.

### 3.4. Impact of Parental Instability on Developmental Outcomes

Finally, we were interested in whether parental instability would have an additive or interactive effect on later child neurodevelopmental outcomes at age 12. Analyses of variance were used to predict socioemotional, behavioural, and educational outcomes at age 12 from children’s gestational status (preterm vs. full term) and history of parental instability (presence or absence). Means and standard deviations for outcome variables are presented in Table 4.

#### 3.4.1. Emotional and Behavioural Adjustment

We used a two-way (2 × 2) analysis of variance to model teacher-rated SDQ scales of emotional symptoms, conduct problems, inattention-hyperactivity, peer relationship problems, prosocial behaviour, and overall behavioural problems from preterm birth and history of parental instability. Preliminary analyses explored socioeconomic status as a covariate (i.e., analyses of covariance); however, SES did not contribute significantly to any of the analyses and was excluded from the final models. Across the six subscales there was an overall pattern of significant main effects of preterm birth and parental instability on outcomes, but no significant interactions between these predictors.

Examination of the main effects for each dependent variable indicated a significant impact of preterm birth on inattention-hyperactivity, peer relationship problems, prosocial behaviour, and overall behavioural adjustment (see Table 5). Parental instability was significantly associated with conduct problems, inattention-hyperactivity, prosocial behaviour, and overall behavioural adjustment (see Table 5).

**Figure 3:**
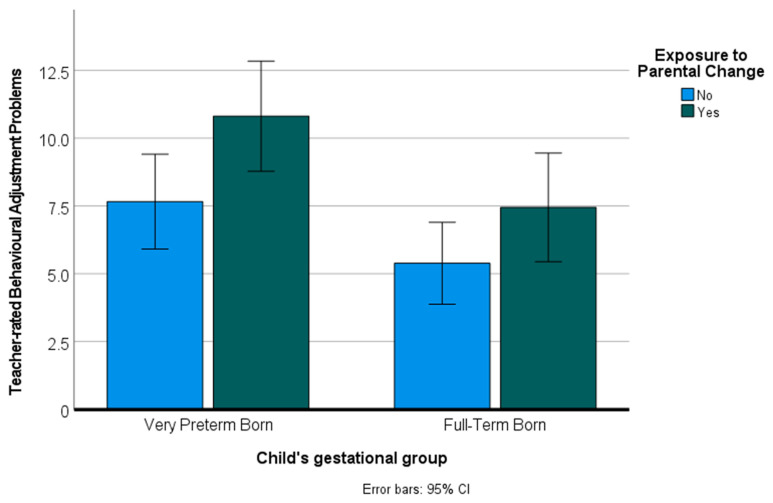
Overall behavioural adjustment based on preterm birth and history of parental instability.

Thus, for inattention-hyperactivity, prosocial behaviour, and overall behavioural adjustment, there was an additive effect of both preterm birth and parental instability. Figure 3 displays the overall behaviour adjustment scores of children based on preterm birth and history of parental instability.

**Figure 4:**
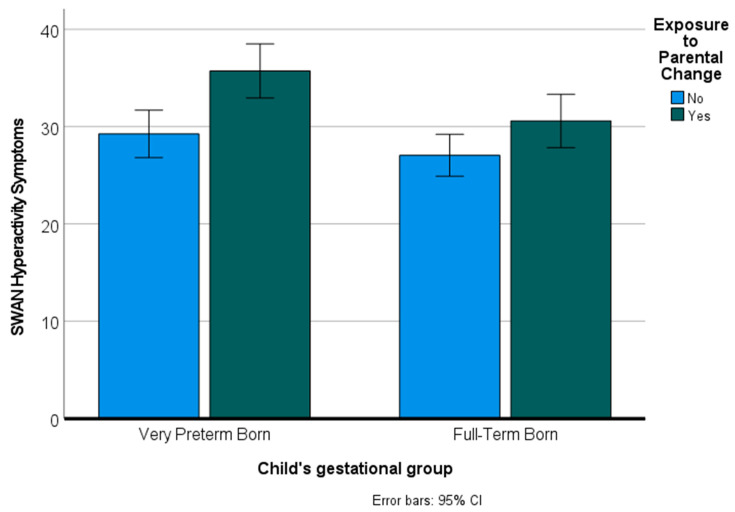
Hyperactivity symptoms based on preterm birth and parental instability.

In addition to SDQ scores, we examined the impact of preterm birth and exposure to parental change on symptoms of attention-deficit/hyperactivity disorder (ADHD). We used a multivariate analysis of variance to predict the two dependent variables of inattention and hyperactivity from preterm birth and parental instability. The overall multivariate tests (Pillai’s Trace) indicated significant main effects for preterm birth [*F*(2160) = 5.03, *p* = 0.008] and parental instability [*F*(2160) = 7.77, *p* = 0.001] but no interaction effect [*F*(2160) = 1.00, *p* = 0.37]. Examination of the effects for each of the dependent variables indicated significant effects of preterm birth and parental change on both inattention symptoms and hyperactivity symptoms [all *F*’s (1161) > 8.13, *p*’s < 0.005]. Figure 4 shows mean hyperactivity symptom scores for VPT and FT children who were exposed and not exposed to parental change.

#### 3.4.2. Cognitive Ability

To understand the impact of preterm birth on educational outcomes we ran an analysis of variance predicting WISC-IV full-scale estimated IQ from preterm birth and parental instability. There was a significant main effect of both preterm birth [*F*(1188) = 20.13, *p* < 0.001] and parental instability [*F*(1188) = 8.80, *p* = 0.003] on full-scale estimated IQ scores, but no interaction between these predictors [*F*(1188) = 2.06, *p* = 0.15]. Preterm birth and parental instability were both predictive of lower cognitive ability at age 12.

#### 3.4.3. Educational Outcomes

To understand the impact of preterm birth on educational outcomes we ran analyses of variance predicting the outcomes of WJ-III broad math standard scores and WJ-III broad reading standard scores from preterm birth and parental instability. When predicting broad math skills, there was a significant main effect of both preterm birth [*F*(1186) = 23.06, *p* < 0.001] and parental instability [*F*(1186) = 4.21, *p* = 0.04], but no interaction between these predictors [*F*(1186) = 0.02, *p* = 0.88]. A similar pattern was seen for broad reading skills, with significant main effects for preterm birth [*F*(1186) = 8.59, *p* = 0.004] and parental instability [*F*(1186) = 4.71, *p* = 0.03] but no interaction effect [*F*(1186) = 2.49, *p* = 0.12]. The results indicate an additive effect whereby children are doubly disadvantaged if they are born preterm and have a history of parental instability.

## 4. Discussion

This paper examined the longer-term impacts of very preterm birth on the partner relationships of parents of children born very preterm. Specifically, we examined whether very preterm birth may increase the likelihood that children will experience parental change/instability over their first 12 years, and in particular whether risks varied by infant gestational age at birth. Further, we examined potential risk factors for parental change and assessed the impact of this parental change/instability on children’s outcomes at age 12, above and beyond risks associated with preterm birth itself.

Results indicate that very preterm birth is associated with an increased risk of parental change/instability from birth to age 12 years. These changes included parental divorce and separation, formation of new parental partner relationships, and adoption or foster care. There was a general tendency for the risk of any and multiple parental changes to be greatest for those born EPT, or those at the lower limits of viability. While children born extremely preterm had higher rates of both any and multiple parental changes than children born full term, children born very preterm were more likely than FT-born children to experience multiple or high levels of instability (two or more parental changes). By corrected age 12 years, nearly half of our sample of children born extremely preterm had experienced at least one parental change, and nearly a third had experienced two or more parental changes.

### 4.1. Preterm Birth as a Predictor of Parental Instability

Consideration of the predictors of parental change within the group of children born either very or extremely preterm showed that, in addition to gestational age at birth, early neonatal medical complexity was associated with increased risk of later parental instability at ages 9 and 12, suggesting longer-term impacts. Research on the impact of medical conditions such as autism spectrum disorder and ADHD has shown an extended period of the risk of divorce, persisting beyond early childhood and into early adulthood [49]. Our results demonstrate a similar picture, whereby medical complexity associated with preterm birth may result in continued parenting stress and unique demands placed on families beyond early childhood and into the school years. In the general population, divorce and separation are most common during the early childhood years [50], and thus the impacts of preterm birth and other health conditions may become more evident once the rates of instability decline in the population of families of typically developing children. We did find that the number of child neurodevelopmental impairments predicted parental instability at age 2, and this finding in combination with the large (non-significant) odds ratio for neonatal medical complexity at age 2 suggests there may be an initial impact of medical complexity/impairment on parental stability in the two years following preterm birth in addition to the longer-term impact described above.

We found that low SES was a significant predictor of parental instability at all ages. This is consistent with previous research showing that instability is more common within low SES families [51]. Families living with the hardships and social conditions associated with low SES may have fewer resources and supports available to them to manage any health or developmental issues that arise as a result of preterm birth. Hospital stays and medical diagnoses can be of significant financial burden to families [18,52], and this burden may be particularly detrimental to those who are already struggling. Financial strain has been linked to a number of aspects of romantic relationships such as emotional distress, disagreements, and reduced quality time, ultimately resulting in marital instability [53]. Thus, increased financial strain is one potential explanation for the increased risk of parental instability due to preterm birth.

Our findings also confirmed previous research suggesting higher rates of parenting stress in parents of children born very preterm, as compared to those born at full term [3,4]. Further, our results are consistent with research showing that decreased marital quality and marital dissolution are more likely when parents are stressed, anxious and depressed [54]. Specifically, our results show that maternal mental health was a significant predictor of parental change/instability within the group of preterm families at ages 6 and 12, with increased symptoms of depression, anxiety, and parental distress associated with the risk of experiencing instability. These findings suggest that longer-term, ongoing mental health issues associated with preterm birth may be associated with family breakdown, particularly as children become older.

Collectively findings suggest that children born at lower gestations, subject to a more complex medical course during their neonatal hospital stay, living in lower socioeconomic conditions, and with mothers experiencing symptoms of depression, anxiety, and/or parenting stress are most at risk of exposure to instability of parents/caregivers.

### 4.2. Neurodevelopmental Outcomes

Finally, we examined the combined effects of preterm birth and parental change/instability on children’s neurodevelopmental outcomes at age 12. For most outcomes assessed, our results indicate an additive effect whereby children were doubly disadvantaged if they were born very preterm and then experienced subsequent parental change/s.

An additive effect (where both preterm birth and parental change uniquely contributed to outcomes) was found for symptoms of inattention and hyperactivity (as assessed by both the SDQ and the SWAN), cognitive ability, educational outcomes, prosocial behaviour, and overall behavioural difficulties on the SDQ. There was an effect of preterm birth but not parental instability on peer relationship problems, and there was an effect of parental instability but not preterm birth on conduct problems. This latter finding is consistent with previous research that has found an inconsistent or lack of association between preterm birth and later conduct problems [55].

These findings show that the family psychosocial context, in terms of maternal mental health, caregiver stability, and socioeconomic status, has long-term implications for the developmental outcomes of children born preterm. Strains on inter-partner relations and family stability may be greatest in families caring for a medically and neurodevelopmentally complex child, and in a context of limited financial and mental health/parenting resources and parenting/mental health stress. Taken together, our findings suggest a negative feedback loop in many families of children born very preterm, whereby the medical burden of preterm birth contributes to family breakdown and instability, which in turn negatively impacts children’s neurodevelopmental outcomes.

Our results thus highlight the need for family-focussed neonatal and postnatal care strategies to support parents as well as their infants to ensure optimal child health outcomes. Monitoring of family and environmental factors ought to be a key component of neonatal and long-term follow-up. This is important as strategies to prevent parental and familial instability following preterm birth, such as support and resources directed at parental mental health needs and supporting their interparental relationships, will help to protect children from experiencing compounded risk to their cognitive, educational, and behavioural development.

### 4.3. Limitations and Future Directions

Our research focused on parental change and therefore does not take into account other types of family instability or disruption that children may experience. The comings and goings of other household members such as grandparents, other adult relatives and non-relatives, or siblings may also impact family functioning and child outcomes, particularly when household members are frequently changing. We chose to focus on parental change as it is likely of primary importance to child developmental outcomes and also most likely to be impacted by preterm birth. We also did not differentiate between the types of parental instability when examining outcomes. There may be differential effects based on the type of change experienced (e.g., parental separation vs. introduction of a new partner) but we did not have a large enough sample to explore the specific patterns associated with each type of parental change.

Finally, in our consideration of psychosocial family factors, we focused on mothers (e.g., maternal age, maternal mental health) and have not examined the psychological well-being of fathers. Further work is required to better understand the contribution of paternal factors to parental instability in the families of children born very preterm.

### 4.4. Conclusions

Our results indicate that preterm birth is associated with an increased risk of parental change/instability over the first 12 years of life. The risk of parental change was greatest for those born extremely preterm, with EPT children being 1.7 times more likely than FT-born children to experience any parental change and twice as likely to experience multiple (2 or more) parental changes. Neonatal medical complexity, family socioeconomic disadvantage, maternal psychological wellbeing, and concurrent child neurodevelopmental impairment were also associated with an increased likelihood of parental change. Furthermore, preterm birth and exposure to parental change/instability both contributed negatively to outcomes (behavioural adjustment, ADHD symptoms, educational and cognitive ability) in an additive, rather than an interactive, manner. These findings support the importance of family-focussed neonatal and postnatal care strategies to support parents as well as their infants for optimal child health outcomes.

## Figures and Tables

**Table 1:** Characteristics of the sample.

	EPT (*n* = 38)	VPT (*n* = 48)	FT (*n* = 111)	F/χ^2^	*p*
Infant clinical factors					
Gestational age, M (SD)	25.5 (1.5) ^a^	29.4 (1.3) ^b^	39.6 (1.1) ^c^	2312.79	<0.001
Birth weight, M (SD)	822 (229) ^a^	1261 (233) ^b^	3597 (396) ^c^	1401.79	<0.001
Male%	50.0% ^a^	60.4% ^a^	53.2% ^a^	1.07	0.59
Medical complexity%	92.3% ^a^	35.3% ^b^	-	29.93	<0.001
Family social background					
Maternal age at birth, years, M (SD)	30.8 (6.1) ^a^	30.5 (4.8) ^a^	31.0 (4.4) ^a^	0.14	0.87
Low family SES, M (SD)	3.45 (1.54) ^a,b^	3.71 (1.69) ^a^	2.93 (1.36) ^b^	5.20	0.006
Single parent at birth%	26.3% ^a^	12.5% ^a^	11.9% ^a^	4.89	0.09
Low maternal education% ^1^	36.8% ^a^	41.7% ^a^	19.3% ^b^	10.00	0.007
Maternal ethnicity					
Māori	5.1% ^a^	5.9% ^a^	5.4% ^a^	0.03	0.99
NZ/other European	87.2% ^a^	92.2% ^a^	92.8% ^a^	1.21	0.55
Pacific Island	2.6% ^a^	0.0% ^a^	1.8% ^a^	1.15	0.56
Asian	2.6% ^a^	2.0% ^a^	0.0% ^a^	2.57	0.28
Other	2.6% ^a^	0.0% ^a^	0.0% ^a^	4.18	0.12
Maternal well-being (Age 2)					
HADS depression score, M (SD)	6.00 (2.47) ^a^	6.48 (2.53) ^a^	6.92 (2.79) ^a^	0.89	0.41
HADS anxiety score, M (SD)	7.26 (2.63) ^a^	7.12 (2.65) ^a^	5.82 (1.89) ^b^	3.78	0.03
Parenting stress score, M (SD)	22.91 (9.13) ^a^	23.09 (7.42) ^a^	24.45 (6.45) ^a^	0.41	0.66

Note: characteristics are provided for the families with data available at the Age 2 assessment; differing superscripts indicate significant differences between gestation groups (i.e., contrasts within the same row); ^1^ Low maternal education was defined as whether the mother had not graduated high school.

**Table 2:** Rates of parental change by gestational group.

Age	Full Term (GA 37+ Weeks)	Very Preterm (GA 28–32 Weeks)	Extremely Preterm (GA < 28 Weeks)
	Any parental change		
2	7.2%	7.8%	18.0%
4	12.6%	15.7%	30.8%
6	23.4%	21.6%	41.0%
9	31.5%	25.5%	46.2%
12	36.0%	37.3%	48.7%
Overall	22.2%	21.6%	36.9%
	Two or more parental changes		
2	2.7%	5.9%	5.1%
4	5.4%	11.8%	18.0%
6	7.2%	15.7%	23.1%
9	15.3%	23.5%	30.8%
12	22.5%	25.5%	30.8%
Overall	10.6%	16.5%	21.5%

GA = gestational age.

**Table 3:** Odds ratios for predictors of any parental instability in children born preterm.

Logistic Regression Odds Ratios					
	Age 2	Age 4	Age 6	Age 9	Age 12
Medical Risk Factors					
Gestational age (weeks)	0.70	0.73	0.55 **	0.65 **	0.70 *
Medical complexity	6.15	1.79	1.58	3.83 *	7.83 **
Family Psychosocial Risk Factors					
Low SES	1.65 *	2.08 **	2.28 **	1.74 **	1.68 **
Maternal age	1.14	0.97	1.07	1.01	0.93
Maternal mental health	1.57	1.06	1.87 **	1.13	1.76 **
Child Risk Factors					
Child impairments	1.34 *	1.21	1.19	1.08	1.16

* *p* < 0.05; ** *p* < 0.01.

**Table 4:** Means and standard deviations for developmental outcomes at age 12.

	EPT (*n* = 38)	VPT (*n* = 48)	FT (*n* = 106)	*F*	*p*
SDQ total difficulties	10.45 (8.12) ^a^	7.75 (5.67) ^a,b^	6.12 (5.88) ^b^	6.60	0.002
SWAN inattention	37.47 (11.44) ^a^	33.28 (8.86) ^a,b^	30.28 (9.22) ^b^	6.81	0.001
SWAN hyperactivity	34.16 (10.42) ^a^	30.60 (7.62) ^a,b^	28.40 (7.93) ^b^	5.63	0.004
IQ	95.56 (15.52) ^a^	98.61 (15.37) ^a^	106.67 (13.65) ^b^	10.32	<0.001
Broad reading	99.14 (20.98) ^a,b^	98.92 (18.12) ^a^	105.71 (14.52) ^b^	3.72	0.026
Broad math	86.33 (18.24) ^a^	90.08 (15.85) ^a^	100.95 (17.19) ^b^	13.03	<0.001

Note: Differing superscripts denote significant differences between gestational groups.

**Table 5:** Main effects of preterm birth and parental instability on strengths and difficulties.

	*F*-Statistic	*p*-Value
Main effect: Gestational Group (preterm vs. full term)		
Emotional symptoms	2.27	0.13
Conduct problems	2.74	0.10
Inattention-hyperactivity	7.49	**0.007**
Peer relationship problems	8.68	**0.004**
Prosocial behaviour	5.21	**0.02**
Overall behavioural adjustment	9.16	**0.003**
Main effect: Parental instability (presence vs. absence)		
Emotional symptoms	1.08	0.30
Conduct problems	9.55	**0.002**
Inattention-hyperactivity	10.55	**0.001**
Peer relationship problems	1.02	0.31
Prosocial behaviour	14.54	**<0.001**
Overall behavioural adjustment	7.83	**0.006**

## Data Availability

The data presented in this study are available on request from the corresponding author.
